# Magnetic interactions in the Zn-Co-O system: tuning local structure, valence and carrier type from extremely Co doped ZnO to ZnCo_2_O_4_

**DOI:** 10.1038/srep16863

**Published:** 2015-11-18

**Authors:** Bastian Henne, Verena Ney, Katharina Ollefs, Fabrice Wilhelm, Andrei Rogalev, Andreas Ney

**Affiliations:** 1 Institut für Halbleiter- und Festkörperphysik, Johannes Kepler Universität, Altenberger Str. 69, 4040 Linz, Austria; 2 ESRF-The European Synchrotron CS40220, 38043 Grenoble Cedex 9, France

## Abstract

We have investigated the relation between local structure, valence and carrier type with magnetism in the Zn-Co-O system. Thin films ranging from wurtzite Zn_1−x_Co_x_O (Co:ZnO) to ZnCo_2_O_4_ spinel were grown on *c*-sapphire substrates. On the one hand, the unprecedented doping of x = 0.6 Co in ZnO enables to study the structural and magnetic properties well-above the coalescence limit. On the other hand, the ZnCo_2_O_4_ spinel provides a *p*-type environment. We find a strong correlation between local structure, valence and carrier type throughout the Zn-Co-O system. In contrast to earlier publications neither 60% Co:ZnO nor ZnCo_2_O_4_ exhibit any sign of ferromagnetic order despite of the high concentration of magnetic ions and a *p*-type carrier background. Instead, antiferromagnetic exchange is found to be the predominant magnetic interaction in the Zn-Co-O system.

Oxide semiconductors and especially doped ZnO have attracted considerable attention over the last decade because of the variety of possible applications. For example, Al-doped ZnO is an attractive conducting oxide material for transparent electronics^1^ and even ZnO-based light emitting diodes were reported^2^. Oxide-based dilute magnetic semiconductors (DMS) and especially ZnO doped with transition metal ions like Co (Co:ZnO) have been studied since decades, *e.g., Refs.*^3^,^4^. The prospect to realize a ferromagnetic DMS at room temperature based on Co:ZnO^5^ has renewed the interest in Co:ZnO. Over the last decade, however, the reproducibility and consistency of the reported magnetic properties of Co:ZnO remained unsatisfyingly controversial. In some cases the observed magnetism could be ascribed to extrinsic effects like the formation of secondary (metallic) Co phases using element specific magnetometry based on X-ray magnetic circular dichroism (XMCD)^6^,^7^. Theory has invoked many potential mechanisms for robust magnetic order in Co:ZnO two out of which so far have rarely been pursued by experimental investigations: On the one hand, doping above the coalescence limit was reported to induce long-range magnetic order in Co:ZnO^8^. On the other hand, the experimental challenge of achieving *p*-type conductivity in Co:ZnO makes it difficult to verify the theoretical predictions of carrier-induced ferromagnetism^9^. In general, Co:ZnO is a hexagonal wurtzite material in which the cobalt atoms are substitutionally incorporated as Co^2+^ on tetrahedrally coordinated Zn lattice sites (Co_*Zn*_). The good dopability of ZnO with Co has already enabled doping concentrations of 20% without phase separation^10^ – the coalescence limit of the cationic sublattice, above which continuous Co paths exist throughout the sample^11^. Even higher concentrations up to 40% were realized via a digital alloy, *i.e.*, a ZnO/CoO multilayer^12^. A further increase of the Co concentration to 60% would relate this extremely doped Co:ZnO to the ZnCo_2_O_4_ spinel, which is under discussion as anode material for lithium batteries^13^ and as a *p*-type gate in junction field-effect transistors^14^. ZnCo_2_O_4_ is a known *p*-type conducting oxide material in which Co is incorporated at octahedral sites (Co_*Oh*_) in a formal valence state of Co^3+^, while the Zn is located at tetrahedral sites as Zn^2+^ (Zn_*Th*_)^15^. Zn_*Oh*_ antisite defects are suspected to act as active acceptors which produce the holes responsible for the observed *p*-type conductivity^16^. In addition, ZnCo_2_O_4_ was reported to behave as hole-mediated ferromagnetic semiconductor^17^,^18^. It has even been held responsible for the observed ferromagnetism in some Co:ZnO DMS samples as a potential secondary phase^19^. However, the relation between local structure, valence and carrier type with the resulting magnetic properties of the Zn-Co-O system from the highly doped wurtzite DMS Co:ZnO to the ZnCo_2_O_4_ spinel end of the compositional range was never studied before, although it would offer the possibility to study the perspectives for both, coalescence *and* hole-mediated ferromagnetism throughout the entire Zn-Co-O system.

Here, we present a systematic study of the evolution of the local structural properties, resulting valence and carrier type from 60% Co:ZnO to ZnCo_2_O_4_ and its interrelation with the magnetic properties. Across the entire Zn-Co-O system antiferromagnetic interactions are consistently being found by high-field XMCD magnetometry, irrespective of the Co being Co^2+^ in tetrahedral coordination and highly resistive, or Co^3+^ in octahedral coordination and *p*-type. Remarkably, the entire sample series could be grown from one single mixed-oxide target using reactive magnetron sputtering and solely varying the preparation conditions.

## Experimental Details

A series of Zn-Co-O epitaxial films with a nominal thickness of 200 nm was grown by reactive magnetron sputtering on polished *c*-sapphire substrates under ultra high vacuum (UHV) conditions (*p* ~ 2 × 10^−9^ mbar) using a stoichiometric mixed oxide composite target (ZnO : Co_3_O_4_ 3 : 2). The Ar : O_2_ ratio was systematically varied by individual mass-flow controllers from 10 : 0 to 10 : 2 standard cubic centimeter per minute (sccm) while the process pressure was kept constant at 4 × 10^−3^ mbar. The growth temperature was changed from room temperature to 525 °C.

The resulting crystal quality was investigated by X-ray diffraction (XRD) using a PANalytical X’Pert Pro MRD to record *ω*/2*θ* and *ω*-rocking scans. X-ray absorption near edge spectroscopy (XANES) measurements were conducted at beamline ID12 at the European Synchrotron Radiation Facility (ESRF) in Grenoble under 15° grazing incidence using total fluorescence yield detection^20^. The X-ray linear dichroism (XLD) was derived by taking the direct difference of two normalized XANES recorded with two orthogonal linear polarizations at 300 K. A quarter wave plate was used to flip the linear polarization of the synchrotron light from vertical to horizontal, *i.e.*, from parallel to perpendicular to the surface normal of the film. The XMCD was taken as the direct difference of XANES recorded with right and left circular polarized light at 2 K and 17 T. To minimize artifacts the direction of the external magnetic field was reversed as well. Element-selective magnetization curves at 2 K were recorded by flipping the circular polarization at fixed photon energies in magnetic fields of up to 17 T. Integral magnetization measurements at 2 K were performed using a commercial SQUID magnetometer (Quantum Design, MPMS-XL5) applying up to 5 T in the film plane.

## Results and Discussion

**Figure 1:**
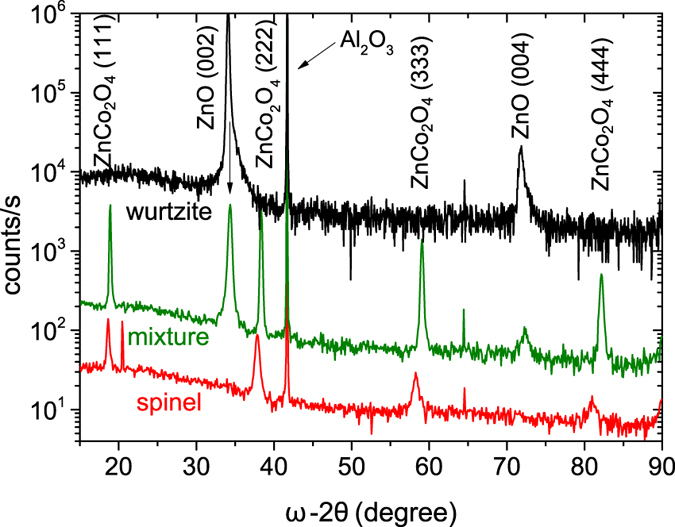
X-ray diffractograms of three representative Zn-Co-O sample species (spinel [red], mixed [green] and wurtzite [black]) (curves shifted for clarity).

Figure 1 depicts the *ω*/2*θ* X-ray diffractograms of three representative types of Zn-Co-O species which are obtained throughout the growth series. (i) specimens for which only the (Co:)ZnO (002) reflection (and higher orders) is present (“wurtzite”, black line). (ii) specimens which only exhibit the ZnCo_2_O_4_ (111) reflection and higher orders (“spinel”, red line). (iii) most samples of the series contain XRD reflections of both crystal structures, namely the wurtzite and the spinel reflections (“mixture”, green line). Besides the reflections from the *c*-sapphire substrate no further reflections are observed in any sample of the series. Disregarding for now the potential existence of amorphous phases, the XRD results indicate that it is possible to obtain both: pure wurtzite Co:ZnO and pure cubic ZnCo_2_O_4_ as well as samples where both phases are mixed. Energy dispersive X-ray (EDX) measurements indicate a cobalt content of 60% for the wurtzite sample, which lies distinctly above the coalescence limit of about 20%^11^ and is to the best of our knowledge the highest attained Co concentration in Co:ZnO so far. Surprisingly, the crystal retained the ZnO wurtzite structure instead of forming a CoO-like cubic lattice.

**Figure 2:**
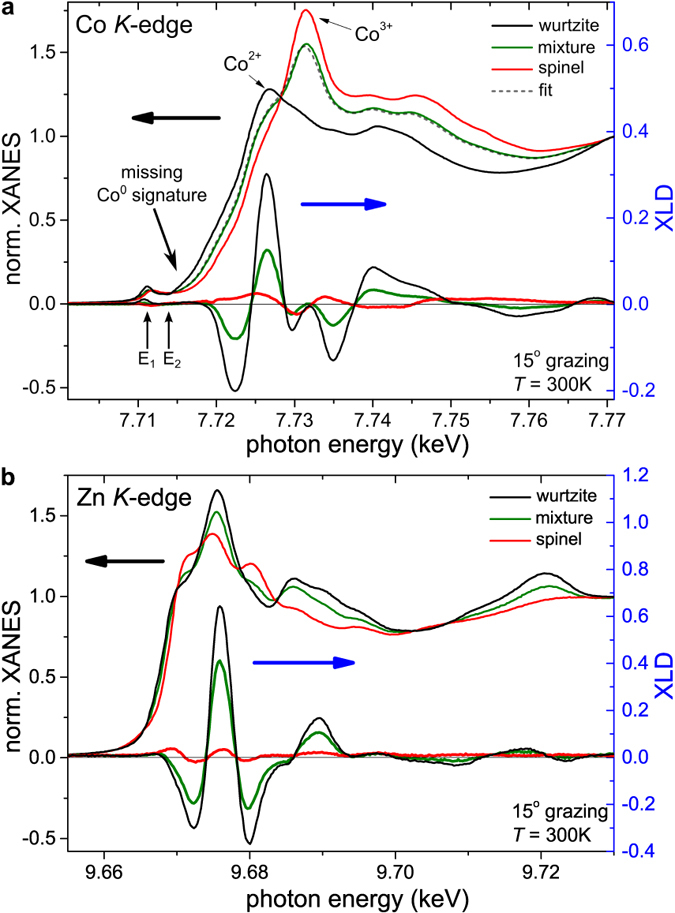
XANES and XLD spectra recorded at the Co (**a**) and Zn (**b**) *K*-edge for the 60% Co:ZnO (black), spinel (red) and mixed phase (green). For Co^3+^ the absorption edge shifts to higher energies compared to Co^2+^. The XANES of the mixed sample is fitted via two reference spectra (see text). At the Zn-edge the main feature remains at the same position, but a shoulder appears. The size of the XLD signal decreases in a similar way for both systems.

A more thorough investigation based on XANES measurements offers the possibility to probe the local structure and valency with element selectivity. A meaningful set of reference spectra and quality indicators has already been established for Co:ZnO^6^. Additionally, reference spectra for ZnCo_2_O_4_ are already available. This allows to assure phase-pureness beyond XRD because the spectral shape of the XANES is sensitive to the respective oxidation states of Co^7^ and does not depend on whether the studied samples are crystalline or amorphous. Figure 2(a) depicts the XANES at the Co *K*-edge for the three representative samples shown in Fig. 1. First of all we want to note that the XANES of the Co *K*-edges of the wurtzite and the spinel samples are virtually identical to the respective references of the phase-pure materials^7^. It is also important to mention that the presence of metallic Co^0^ can be excluded by the existence of a distinct pre-edge feature in all spectra. In particular, the application of the quality indicator for the pre-edge feature for wurtzite Co:ZnO, given by XANES(E_1_)/XANES(E_2_) (compare Fig. 2 with Fig. 2(a) in Ref. 6), yields a value of 1.84 which indicates that the specimen is devoid of metallic Co down to less than 1%.

Turning back to the comparison of the wurtzite and the spinel samples, clear differences between the Co *K*-edge XANES are evident. The onset of the Co main absorption gradually shifts to higher energies by going from the Co:ZnO over the mixed sample to the spinel [Fig. 2(a)]. This so-called chemical shift reveals a change of the valence state from 2+ to 3+ of the incorporated Co cations with increasing spinel content. In addition, also the spectral shape of the main absorption changes and the maximum shifts from 7.726 keV (wurtzite) to 7.731 keV (spinel). These changes in the Co *K*-edge XANES are thus characteristic for the change from Co^2+^ in ZnO to Co^3+^ in ZnCo_2_O_4_. The Co *K*-edge spectrum of the mixed sample contains features of both Co species. To quantitatively determine the Co^2+^ content in this mixed sample the Co *K*-edge spectra was fitted by a weighted superposition of the two reference spectra (*x* × XANES[Co:ZnO] + (1 − *x*) × XANES[ZnCo_2_O_4_]). Since the fitted spectrum matches the measured data very well [Fig. 2(a) (dashed line)], this yields a Co^2+^ content of 39% within a few percent accuracy.

The XANES at the Zn *K*-edge also shows clear changes in the fine-structure of the main absorption for the three representative samples [Fig. 2(b)]. However, the edge essentially remains at the same energy position indicating that Zn remains in its formal 2+ oxidation state. The small shift of the absorption edges’ inflection point towards higher energies in the spinel system may be attributed to the presence of a small amount of the antisite defect (Zn_*Oh*_ and thus Zn^3+^) which – according to ref. 16 – would favor *p*-type behavior in this spinel sample.

The corresponding XLD at the Co and Zn *K*-edges is shown in Fig. 2 as well (right scales). In uniaxial crystals like wurtzite ZnO the size of the XLD is a measure for the degree of local structural anisotropy and by comparing it with simulations one can quantify the amount of substitutional incorporation, *e.g.*, of Co on Zn lattice sites in ZnO^21^. Therefore, we first focus on the 60% Co:ZnO sample where an XLD amplitude of 0.5 and 0.95 is measured at the Co and the Zn *K*-edges, respectively. This is slightly lower than the reference values of 0.67 and 1.11 for phase-pure Co:ZnO which were derived in Ref. 6, 7. However, one has to note that the films grown in this series have a slightly reduced crystalline quality and increased mosaicity as indicated by the full-width at half-maximum (FWHM) in XRD *ω*/2*θ* and *ω*-rocking scans accounting for a slight reduction of the XLD at both edges. In addition, the effective Co thickness is considerably higher due to the 6-fold higher Co concentration present in the specimens. Therefore, self-absorption plays a more important role, leading to a further decrease of the XLD at the Co *K*-edge. Nonetheless, one can assert by the size of the XLD that the Co atoms are almost exclusively incorporated on Zn lattice sites in the ZnO matrix (Co_*Zn*_) despite the extremely high doping level of 60%. On the other hand, Fig. 2(a) reveals that the XLD amplitude at both edge decreases for the mixed sample, yet maintaining its spectral shape. Finally, it reduces as expected to almost zero with a significantly altered residual spectral shape in the cubic spinel sample. The latter is accounted for by the lack of structural anisotropy of the cubic system and thus corroborates the findings of XRD in Fig. 1 on a local scale.

**Figure 3:**
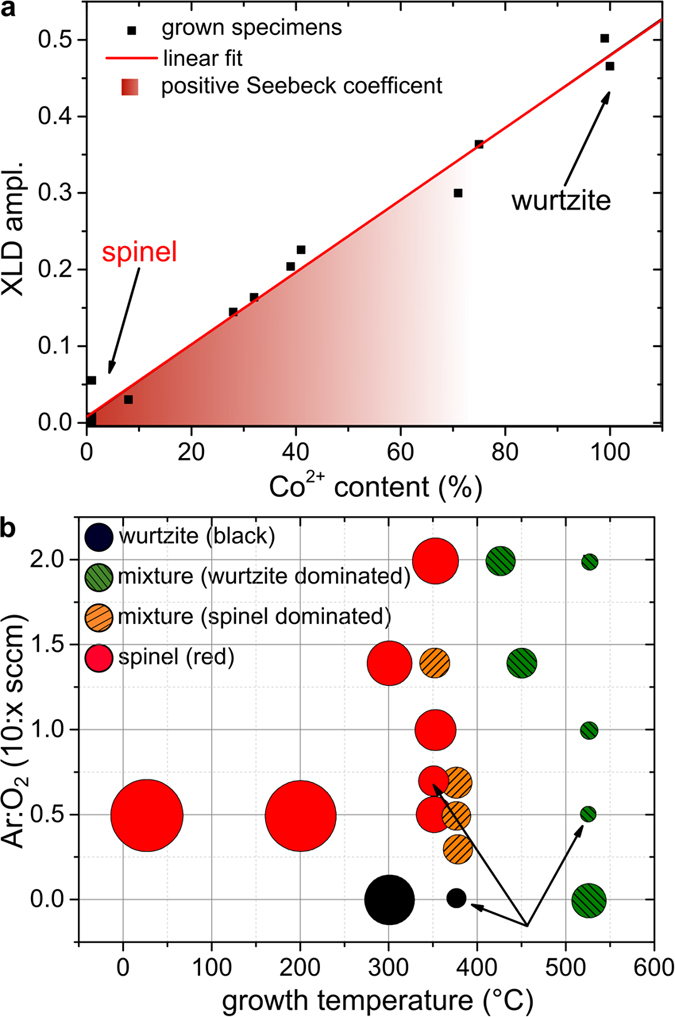
(**a**) XLD amplitude as a function of the Co^2+^ content as obtained by superposition of reference spectra for all the samples of the series (see text). (**b**) Parameter space map of the produced samples. The dot size represents FWHM of the spinel reflection in XRD. The color indicates if only the spinel reflection (red), the wurtzite reflection (black) or both (orange/green) are present.

Obviously, the loss in local crystalline anisotropy can be quantified by the reduction of the Co XLD while the change in valence can be derived by the aforementioned fit procedure of the Co *K*-edge XANES. Across the growth series this is accompanied by a change from tetrahedral to octahedral coordination of the Co atoms given by the change in the global crystal structure. In Fig. 3(a) the XLD amplitude of the Co *K*-edge as a measure of the amount of Co in a wurtzite ZnO environment is plotted against the Co^2+^ content derived by the valence-selective fitting procedure of the XANES. It is obvious that the XLD amplitude and thus the local symmetry, *i.e.*, tetrahedral coordination in a wurtzite environment scales linearly with the valence, *i.e.*, the Co^2+^ content. This one-to-one correlation of the local structure and the valence is another indication for the (sub-)phase-pureness of the produced samples and underlines that Co^3+^ is not found in a tetrahedral/wurtzite (ZnO) environment. In turn, its presence is therefore strictly linked to the presence of the octahedral/cubic phase of the Zn-Co-O system. Therefore, a strong correlation between local structure and valence can be established for the Zn-Co-O system across the entire growth series. However, the spatial distribution of both subphases in the mixed samples cannot directly be determined. The fact that atomic force microscopy reveals a rather smooth surface with a root mean square roughness of less than 2 nm (not shown) together with the absence of XRD reflections of other crystalline orientations beyond ZnO (002) and ZnCo_2_O_4_ (111) indicates rather homogeneously mixed coexisting crystallites of the two subphases.

The low mobilities and the high resistance of our samples impeded reliable Hall effect measurements throughout the series. Therefore, Seebeck measurements have been carried out to determine the dominant carrier type in each specimen. All samples which contain a dominant fraction of the spinel phase consistently show positive signs of the Seebeck voltage, confirming the *p*-type character as indicated by a shaded area in Fig. 3. The mixed samples are prevalently *p*-type as well, while the pure wurtzite samples are highly resistive (*ρ* > 85 kΩ cm) and thus no conclusive sign of the Seebeck coefficient could be determined. However, usually these types of highly resistive Zn_1−*x*_Co_*x*_O samples are found to be *n*-type^22^. Obviously, the *p*-type character of the spinel subphase dominates over in intrinsic background *n*-type character of the wurtzite subphase in the mixed system. In turn this infers that in the mixed samples Co:ZnO exists in a *p*-type carrier background. Note however, that this does not necessarily mean that the Co:ZnO subphase itself is actually *p*-type in these samples. These findings, nonetheless, stand to reason that the observed carrier type is closely linked to the local structure and thus the valency of the Co.

Figure 3(b) finally depicts the preparation parameter space map of the whole growth series, indicating which growth temperatures and Ar : O_2_ ratios in the sputter gas favor which phase. To estimate the crystalline quality of the respective films the size of the dots represents the FWHM of the ZnCo_2_O_4_ (222) reflection. If the sample does not contain any detectable ZnCo_2_O_4_ in XRD, the FWHM of the Co:ZnO (002) reflection was used. The color coding of the dots classifies the predominant phase in the sample. If only the spinel reflection is present, the dot is marked red; if only wurtzite reflections are observed it is black. If both phases are detectable, a tendency to the prevalent species (via the intensity of the reflection) is given (orange → spinel / green → wurtzite). The specimens discussed in Figs 1 and 2 are indicated by arrows. A clear tendency is found that for growth temperatures below about 400 °C the spinel phase is likely to form if additional oxygen is present in the sputter gas, whereas for higher temperatures or the absence of oxygen in the sputter gas the wurtzite phase appears. It is noteworthy that the crystal quality, esp. of the ZnCo_2_O_4_, as observed by the FWHM in XRD becomes better close to the “transition temperature” at around 400 °C. Therefore, the preparation should be performed around this temperature in order to obtain the best possible results. For bulk crystals under ambient pressure it is known that a decomposition from Co_3_O_4_ to CoO occurs at ~800 °C^23^,^24^,^25^. However, it is not unusual that off-equilibrium conditions like epitaxial growth on substrates in UHV may shift such types of decomposition in temperature. In qualitative agreement with the behavior of bulk cobalt oxides, the ZnCo_2_O_4_ phase and thus the existence of Co^3+^ is only stable at lower temperatures under oxidizing conditions.

**Figure 4:**
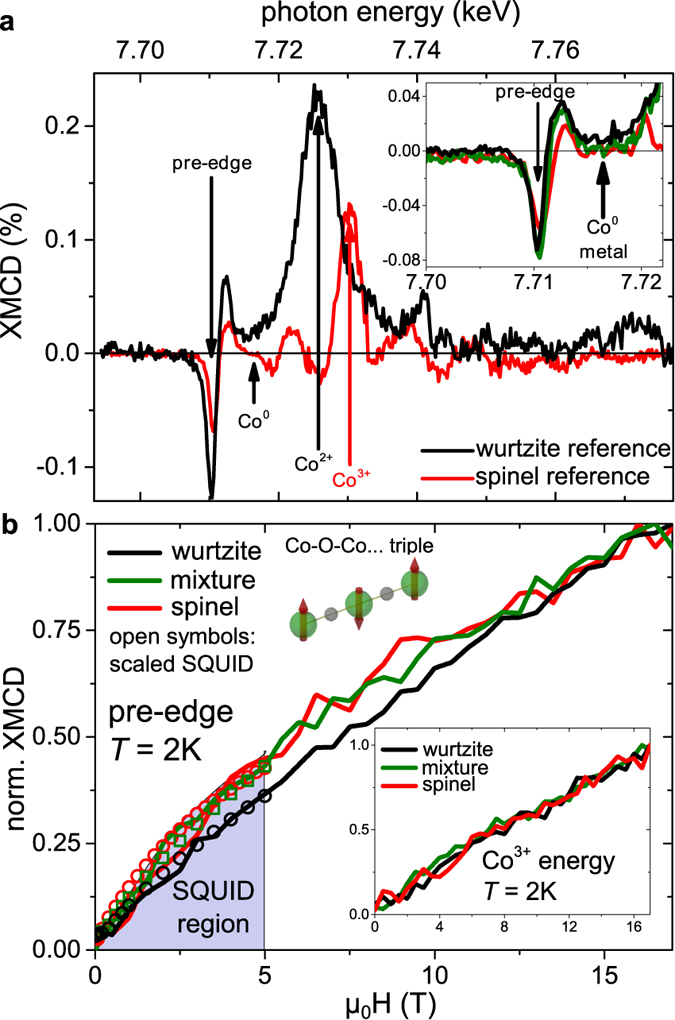
(**a**) XMCD spectra of a wurtzite Co:ZnO and a ZnCo_2_O_4_ reference sample. Features of the individual valencies are marked at which the respective XMCD(*H*) can be recorded. Inset: XMCD spectra for the three representative samples under investigation around the pre-edge. (**b**) XMCD(*H*) curves of the same specimens recorded at the Co pre-edge. Inset: XMCD(*H*) curves recorded at the Co^3+^ energy.

Having established the close interrelation between valence, local structure, and coordination of the Co as well as carrier type in the Zn-Co-O system, the resulting magnetic behavior shall be investigated. In Fig. 4(a) XMCD spectra at 2 K of the Co:ZnO and ZnCo_2_O_4_ reference samples are shown which exhibit a range of well-separated spectral features where valence-specific magnetic information can be probed. At the characteristic pre-edge feature a finite XMCD exists for both Co^2+^ and Co^3+^. Between the pre-edge feature and the main absorption [indicated in Fig. 4(a)] Co metal would have its maximum XMCD signal^6^,^7^. In the inset of Fig. 4(a) it can be seen that all three samples show a clear XMCD at the pre-edge while none of the samples exhibits any sizable XMCD intensity at the photon energy of metallic Co. This demonstrates that no significant amount of metallic Co contributes to the overall magnetic properties thus underlining that our samples are devoid of unwanted secondary phases. Finally, the XMCD of the reference spectra show a distinct fine structure at the main absorption. In Fig. 4(a) two characteristic photon energies are indicated. One allows to exclusively probe Co^2+^ while the maximum XMCD for Co^3+^ also contains a finite contribution of Co^2+^. We first focus on the XMCD(*H*) curves recorded at the pre-edge at 2 K as shown in Fig. 4(b). These are compared to the magnetization curve measured using integral SQUID magnetometry at 2 K and up to 5 T which were scaled to fit the XMCD data. The functional behavior of the M(*H*) curves of the SQUID matches the element-specific XMCD(*H*) rather well. This indicates that the overall magnetic properties of the samples are sufficiently well represented by the Co specific XMCD measurement where higher magnetic fields are attainable. The XMCD(*H*) curves exhibit no difference between wurtzite, spinel and mixture, besides a slightly reduced curvature for the 60% Co:ZnO which we consider to be insignificant within measurement accuracy. In the inset of Fig. 4(b) the Co^3+^-dominated magnetization curves recorded at 2 K for all three species are shown which are virtually indistinguishable over the entire field range. At the characteristic energy of Co^2+^ identical XMCD(*H*) curves could be recorded except for the ZnCo_2_O_4_ specimen (not shown).

As Fig. 4(b) and its inset reveal, the XMCD(*H*) curves recorded at 2 K consistently show a steady increase of the XMCD(*H*) even up to 17 T for both characteristic photon energies and thus both valence states of the Co for all studied samples. In 5% to 15% Co:ZnO this non-saturating behavior is already known and was used to quantify the antiferromagnetic next-cation-neighbor coupling of Co-O-Co *pairs* and Co-O-Co-O-Co *triples* which was found to be in the range of 10 K to 15 K^26^. An *open triple* is schematically illustrated in Fig. 4(b). However, the fitting procedure in Ref. 26 relies on an effective-spin Hamiltonian and a Heisenberg-type next cation neighbor exchange coupling as well as the abundance of isolated Co atoms (*singles*), *pairs* and *triples* as calculated for a given Co concentration using Behringer’s equations^27^. In the present case of 60% Co doping, however, the sum of the abundances of *singles*, *pairs* and *triples* account for only ~0.002% of all existing Co atoms so that the magnetic behavior is almost exclusively dominated by much larger Co-O-Co-…-configurations. As discussed in Ref. 26 in more detail the magnetic behavior can qualitatively be described in terms of uncompensated antiferromagnetism stemming from frustrated or remaining uncompensated spins of these larger configurations. However, a more quantitative description proves to be hardly possible because of the extremely large number of possible Co-O-Co-…-configurations far above the coalescence limit. Nevertheless, the non-saturating behavior of the XMCD(*H*) curves is an unambiguous experimental evidence that antiferromagnetic coupling between neighboring Co atoms is the dominant magnetic interaction across the entire growth series from the 60% Co:ZnO to the ZnCo_2_O_4_, and the mixed sample. In none of the samples any indication for ferromagnetic order exists, including the SQUID measurements. Obviously, the strong correlation between local structure, valence and carrier type does not extent to the magnetic properties in the Zn-Co-O system which remains antiferromagnetic irrespective of the other physical properties.

## Conclusion

The interplay of local structure, valence and carrier type with magnetism in the Zn-Co-O system was investigated using element selective synchrotron-based techniques for a series of Zn-Co-O epitaxial films from the (*p*-type) ZnCo_2_O_4_ spinel to the (*n*-type) wurtzite Co:ZnO with an unprecedented Co concentration of 60%. In the Zn-Co-O system a clear 1:1 correspondence of the Co^2+^ content as measured using XANES with a local wurtzite environment as obtained by XLD was found. Seebeck measurements revealed *p*-type character for the spinel dominated, *i.e.*, Co^3+^ containing, samples. Despite the close interrelation between local coordination, global structure, Co valence and carrier type, element specific magnetic measurements by XMCD show no significant differences in the magnetic properties. In particular, the Co sublattice magnetization at 2 K does not saturate up to 17 T for any specimen of the series, being indicative of antiferromagnetic interactions between the Co ions. Thus, previous reports of hole-mediated ferromagnetism in ZnCo_2_O_4_^17^,^18^ as well as coalescence-induced ferromagnetic order in Co:ZnO^8^ cannot be corroborated. Note, however, that around 60% a transition to a layerwise antiferromagnet is predicted in^8^. In summary, we find that the local structure has a strong correlation with the valence of the Co atoms and that in turn the valency strongly influences the predominant carrier type in the Zn-Co-O system. The magnetic properties, however, remain unaffected of the former characteristics and show uncompensated antiferromagnetism as predominant coupling mechanism from the (cubic, octahedral Co^3+^, *p*-type) ZnCo_2_O_4_ spinel to the (hexagonal, tetrahedral Co^2+^, *n*-type) wurtzite DMS Co:ZnO even at extreme doping levels.

## Additional Information

**How to cite this article**: Henne, B. *et al.* Magnetic interactions in the Zn-Co-O system: tuning local structure, valence and carrier type from extremely Co doped ZnO to ZnCo_2_O_4_. *Sci. Rep.* **5**, 16863; doi: 10.1038/srep16863 (2015).

## References

[b1] Hagendorfer H. *et al.* Highly transparent and conductive ZnO: Al thin films from a low temperature aqueous solution approach. Adv. Mater. 26, 632 (2014). 24151235 10.1002/adma.201303186

[b2] Son D. I. *et al.* Emissive ZnO-graphene quantum dots for white-light-emitting diodes. Nat. Nanotechnol. 7, 465 (2012). 22635098 10.1038/nnano.2012.71

[b3] Estle T. L. & De Wit M. Paramagnetic Resonance of Co2+ and V2+ in ZnO. Bull. Am. Phys. Soc. 6, 445 (1961).

[b4] Koidl P. Optical absorption of Co 2+ in ZnO. Phys. Rev. B 15, 2493 (1977).

[b5] Ueda K., Tabata H. & Kawai T. Magnetic and electric properties of transition-metal-doped ZnO films. Appl. Phys. Lett. 79, 988 (2001).

[b6] Ney A. *et al.* Advanced spectroscopic synchrotron techniques to unravel the intrinsic properties of dilute magnetic oxides: the case of Co: ZnO. New J. Phys. 12, 013020 (2010).

[b7] Ney A. *et al.* Structural, chemical and magnetic properties of secondary phases in Co-doped ZnO. New. J. Phys. 13, 103001 (2011).

[b8] Nayak S. K., Ogura M., Hucht A., Akai H. & Entel P. Monte Carlo simulations of diluted magnetic semiconductors using ab initio exchange parameters. J. Phys.: Condens. Matter 21, 064238 (2009). 21715940 10.1088/0953-8984/21/6/064238

[b9] Dietl T. A ten-year perspective on dilute magnetic semiconductors and oxides. Nature Mater. 9, 965 (2010). 21102516 10.1038/nmat2898

[b10] Ney A. *et al.* Structure, valence, and magnetism of Co-doped ZnO at the coalescence limit. J. Appl. Phys. 115, 172603 (2014).

[b11] Lorenz C., May R. & Ziff R. M. Similarity of percolation thresholds on the HCP and FCC lattices. J. Stat. Phys. 98, 961 (2000).

[b12] Sawicki M. *et al.* Homogeneous and heterogeneous magnetism in (Zn, Co) O: From a random antiferromagnet to a dipolar superferromagnet by changing the growth temperature. Phys. Rev. B 88, 085204 (2013).

[b13] Sharma Y., Sharma N., Subba Rao G. V. & Chowdari B. Nanophase ZnCo_2_O_4_ as a High Performance Anode Material for LiIon Batteries. Adv. Funct. Mater. 17, 2855 (2007).

[b14] Schein F., von Wenckstern H. & Grundmann M. ZnO-Based n-Channel Junction Field-Effect Transistor With Room-Temperature-Fabricated Amorphous p-Type Gate. Electron Device Letters, IEEE 33, 676 (2012).

[b15] Cossee P. Structure and magnetic properties of Co_3_O_4_ and ZnCo_2_O_4_. Recueil 75, 1089 (1956).

[b16] Perkins J. D. *et al.* Inverse design approach to hole doping in ternary oxides: Enhancing p-type conductivity in cobalt oxide spinels. Phys. Rev. B 84, 205207 (2011).

[b17] Kim H. J. *et al.* Growth and characterization of spineltype magnetic semiconductor ZnCo_2_O_4_ by reactive magnetron sputtering. phys. stat. sol. b 241, 1553 (2004).

[b18] Kim H. J. *et al.* Electrical and magnetic properties of spinel-type magnetic semiconductor ZnCo_2_O_4_ grown by reactive magnetron sputtering. J. Appl. Phys. 95, 7387 (2004).

[b19] Liu Y. & MacManus-Driscoll J. L. Impurity control in Co-doped ZnO films through modifying cooling atmosphere. Appl. Phys. Lett. 94, 022503 (2009).

[b20] Rogalev A., Wilhelm F., Goulon J. & Goujan G. Advanced Instrumentation for X-ray Magnetic Circular Dichroism. Springer Proc. Phys. 151, 289 (2013).

[b21] Ney A. *et al.* Absence of intrinsic ferromagnetic interactions of isolated and paired Co dopant atoms in Zn_1−*x*_Co_*x*_O with high structural perfection. Phys. Rev. Lett. 100, 157201 (2008). 18518145 10.1103/PhysRevLett.100.157201

[b22] Ye S. *et al.* Advanced spectroscopic synchrotron techniques to unravel the intrinsic properties of dilute magnetic oxides: the case of Co:ZnO. Phys. Rev. B 80, 245321 (2009).

[b23] Peiteado M., Caballero A. C. & Makovec D. Thermal evolution of ZnCo_2_O_4_ spinel phase in air. J. Ceram. Soc. Jpn. 118, 337 (2010).

[b24] Perry N. H. & Mason T. O. Phase Equilibria of the Zinc Oxide – Cobalt Oxide System in Air. J. Am. Ceram. Soc. 96, 966 (2013).

[b25] Bazuev G. V., Gyrdasova O. I., Grigorov I. G. & Koryakova O. V. Preparation of ZnCo_2_O_4_ spinel whiskers from zinc cobalt oxalate. Inorganic materials 41, 288 (2005).

[b26] Ney A., Ney V., Wilhelm F., Rogalev A. & Usadel K. Quantification of the magnetic exchange via element-selective high-field magnetometry: Co-doped ZnO epitaxial films. Phys. Rev. B 85, 245202 (2012).

[b27] Behringer R. E. Number of Single, Double, and Triple Clusters in a System Containing Two Types of Atoms. J. Chem. Phys. 29, 537 (1958).

